# Evolution of the Dietary Patterns across Nutrition Transition in the Sardinian Longevity Blue Zone and Association with Health Indicators in the Oldest Old

**DOI:** 10.3390/nu13051495

**Published:** 2021-04-28

**Authors:** Giovanni Mario Pes, Michel Poulain, Alessandra Errigo, Maria Pina Dore

**Affiliations:** 1 Dipartimento di Scienze Mediche, Chirurgiche e Sperimentali, University of Sassari, Viale San Pietro 8, 07100 Sassari, Italy; mpdore@uniss.it; 2 Sardinia Longevity Blue Zone Observatory, 08040 Ogliastra, Italy; 3 Institute for the Analysis of Change in Historical and Contemporary Societies (IACCHOS), Université Catholique de Louvain, 1348 Louvain-la-Neuve, Belgium; michel.poulain@uclouvain.be; 4 Estonian Institute for Population Studies, Tallinn University, 10120 Tallinn, Estonia; 5 Dipartimento di Scienze Biomediche, University of Sassari, Viale San Pietro 43/b, 07100 Sassari, Italy; a.errigo@studenti.uniss.it; 6 Baylor College of Medicine, One Baylor Plaza, Houston, TX 77030, USA

**Keywords:** dietary habits, health indicators, human longevity, Longevity Blue Zone, Sardinia

## Abstract

Health and longevity in humans are influenced by numerous factors, including lifestyle and nutrition. However, the direct relationship between lifetime eating habits and functional capacity of the elderly is poorly understood. This study investigated the dietary changes across nutrition transition (NT) in the early 1960s, in a population located in the Sardinia island known for its longevity, dubbed as “Longevity Blue Zone” (LBZ), as well as the relationship between the dietary patterns and a panel of health indicators. A total of 150 oldest-old participants (89 women and 61 men, age range 90–101 years) living in the LBZ were recruited. Participants were interviewed using validated questionnaires to assess the consumption frequency of common food items, as well as the correlation with self-rated health, comorbidity, affective and cognitive level, physical mobility, disability and anthropometric parameters. Differences between subgroups were evaluated using the Mann-Whitney *U* test for independent samples or the Wilcoxon signed-rank test for paired samples. Correlation analysis was performed by calculating the Spearman correlation coefficient, separately in males and females. Compared to the pre-NT epoch, the consumption of meat, olive oil and fresh fruit slightly increased, while the consumption of lard, legumes and vegetables decreased. A significant association was found between increased olive oil intake across NT and self-rated health (ρ = 0.519), mobility (ρ = 0.502), improved vision (ρ = −0.227) and hearing (ρ = −0.314); increased chicken meat intake and performance in activities of daily living (basic activities of daily living: ρ = 0.351; instrumental activities of daily living: ρ = 0.333). Instead, vegetable consumption showed low correlation with health indicators. A mild increase in meat intake, mostly pastured poultry, is associated with better physical performance in the Sardinian LBZ elders, suggesting that a supply of protein may have been crucial to maintaining adequate functional capacity.

## 1. Introduction

The world’s older population is growing at an ever-faster pace, becoming a serious concern for the society due to increased health care expenditure as a result of expansion of mild over severe disability [1]. It has been estimated that there were 703 million persons aged 65 years or over worldwide in 2019, and they will be 1.5 billion in 2050 [2], provided that the SARS-CoV-2 pandemic will subside [3]. Nearly half of people beyond the age of 60 experience multimorbidity that may heavily impact on their quality of life. The efforts of many researchers and health operators worldwide are therefore intensely oriented towards identifying modifiable risk factors for disability in old age, and the most suitable measures for its prevention [4]. Among the myriad genetic and behavioral factors, lifelong dietary habits are of major importance. For instance, results obtained in animal models give hope to extend longevity by diet optimization [5] and several prospective studies in humans have been conducted to ascertain whether a particular diet can influence the ageing phenotype [6,7]. Among the nutritional patterns that have been consistently associated with health and longevity, the Mediterranean diet occupies a pre-eminent position and represents a true cultural model [8,9]. Moreover, it is in a good agreement with the United Nations’ Sustainable Development Goals [10] and several attempts to translate it to non-Mediterranean populations were apparently successful [11].

Over the last two decades, the discovery of geographic areas, known as “Longevity Blue Zones” (LBZs), characterized by a considerable proportion of inhabitants attaining unusually long lives, and often able to escape old age disability, has attracted the interest of investigators. These populations fuel expectations of singling out the key factors associated with the maintenance of functional capacity during aging [12]. Among these communities, one is located in the innermost area of the Mediterranean island of Sardinia, where the number of age-validated centenarians, especially males, is remarkably high [13]. Although numerous explanatory hypotheses have been advanced in the past decade [14,15], the causes of the LBZ’s exceptional longevity are still poorly understood. Considering that the Sardinian population is a genetic isolate [16] it has been speculated that a relatively limited genetic diversity might have favored, at least in part, the emergence of genetic variants conducive to longer survival [17]. Besides genetic factors, the anthropological and cultural features of this population, largely documented in the literature, are also remarkably homogeneous, resulting from a scarcity of contacts between the islanders and the rest of Europeans over the centuries [18]. Therefore, a satisfying explanation for the widespread longevity of this community—for which the term “population longevity” has been proposed [19]—probably requires the involvement of additional epigenetic, environmental or lifestyle factors and their mutual interactions.

The investigation of the effect of the diet on health and longevity in a given population is complicated by the fact that eating habits change over time. In particular, a striking discontinuity corresponds to the period of nutrition transition (NT), whose model, initially proposed by Barry Popkin [20], is still considered valid with some modifications [21]. According to this model, the traditional diet, usually poor and mostly plant-based, consisting in complex carbohydrates, would be progressively enriched with simple sugars and fatty or salty foods of animal origin [22], thus changing into a less healthy food choice. However, excessive generalization of a complex process may not be always valid, and may not capture the geographical heterogeneity of food patterns in some populations.

The traditional diet of the population living in the Sardinian LBZ has often been described as particularly “healthy” and one of the major determinants of the exceptional longevity of this community [23,24]. However, the impact of NT on the global health of this population has rarely been investigated [25,26], and studies were mostly based on the analysis of aggregate data obtained from public sources [27], and not by directly interviewing people who can still recall their diet over the transitional period. Furthermore, the putative effect of the diet on Sardinian longevity is sometimes modelled by assuming that no spatial-temporal variations have occurred, limiting the possible extent of inference [28]. In the Sardinian LBZ, the NT started at the dawn of the 1960s [27], but proceeded slowly due to the persistence of a rural profile grounded in pastoralism and agricultural activities. Hence, the Sardinian diet has maintained until nowadays some aspects of the pre-transitional epoch.

Long considered as hinged in the Mediterranean food tradition, the LBZ population, however, underwent a progressive change in dietary habits, owing to a relative economic development at the end of the 1950s [14]. These historical trends recommend the use of a longitudinal approach when examining the relationship between diet and health outcomes in the Sardinian LBZ.

Three research questions have been posed in this study: (i) what are the consumption frequencies of foods among the current oldest living persons in this unique community, and how did they change over the decades? (ii) how do the dietary patterns, and their temporal change, relate to the currently measurable health status? (iii) was there any sex-related difference in eating habits before and after NT, and how did sex affect health outcomes?

## 2. Materials and Methods

### 2.1. Study Design and Participants

**Figure 1:**
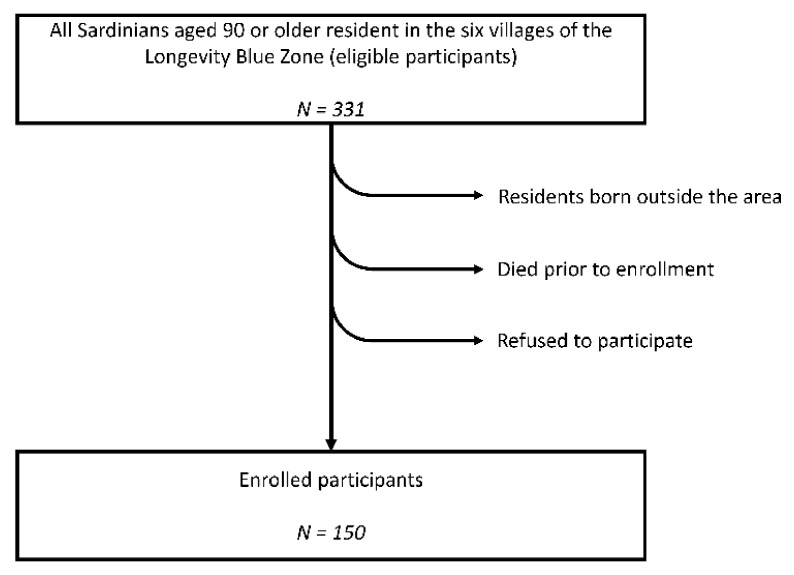
Recruitment process flowchart.

Based on the above premises, the study was specifically designed to (i) describe the dietary pattern currently observable among the oldest LBZ members separately in men and women; (ii) reconstruct the participants’ dietary profile before NT, i.e., at the time of their early adulthood around age 30; (iii) analyze the main dietary changes that occurred between the youth of the interviewees and the present time; (iv) estimate the correlation between the consumption frequency of the main food groups today with health and physical performance indicators, including cognitive and affective status scores, mobility scores, and anthropometric measures. Data were obtained from a sample of the oldest subjects (aged 90–101 years) born and living in the Sardinia LBZ. A self-explanatory flowchart depicting the recruitment process is reported in Figure 1.

All participants were interviewed face-to-face at home by a trained physician (G.M.P.) to collect information on demographics, medical history, health-related behaviors, and dietary habits. When substantial cognitive impairment of the respondent was perceived, a family member was also involved in the interview. Data are part of the Sardinia LBZ study starting in 2018 [29].

### 2.2. Data Collection

Demographic data including age, sex, marital status, education, place of residence, smoking habits and pre-retirement occupation were collected and previously reported [29].

#### 2.2.1. Anthropometric Measures

Body height (m) and weight (kg) were measured in each participant using standard methods [30]. Body mass index (BMI) was expressed in kg/m^2^ [30]. Waist circumference, average calf circumference (measured at the gastrocnemius muscle), average brachial circumference (measured at the biceps muscle) and average leg length were measured in centimeters according to established techniques [31,32]. Anthropometric measures were collected by two of the authors (G.M.P and A.E.)

#### 2.2.2. Multidimensional Geriatric Examination

All study participants underwent a multidimensional geriatric examination at home, using validated questionnaires covering several domains. Medical history included self- and family-reported comorbidity involving any previous disease diagnosis classified according to the Cumulative Illness Rating Scale (CIRS) [33]. More specifically, the illnesses included were: cardiovascular, hypertension, blood, respiratory, eyes-ears-nose-throat-larynx, upper gastrointestinal and pancreas, lower gastrointestinal, liver, kidney, urinary, muscle and bone; central nervous system, endocrine, psychiatric, rheumatic and autoimmune disorders and dementia. In addition, all available medical records were screened and relevant data about illnesses retrieved. Systolic (SBP) and diastolic (DBP) blood pressure was measured multiple times in the left arm using a standard protocol [34]. Blood hypertension was defined as SBP ≥ 140 mm Hg and/or a DBP ≥ 90 mm Hg. The use of antihypertensive medications was considered as a proxy [35]. Hearing and vision were also tested (hearing, by asking the participant to repeat a whispered sentence from a distance of about 4 m; visual acuity, by asking the participant to read or to recognize symbols on an optometric chart with his/her eyes standing at 30 cm from the chart) and results expressed as a 4-levels ordinal variable (1 = normal, 2 = slightly impaired, 3 = severely impaired, 4 = total incapacity).

#### 2.2.3. Performance-Based Functional Capacity

Self-rated health was evaluated according to Idler at al. [36]. Functional capacity was assessed using a six-item basic activities of daily living (BADL) score, by direct questioning of participants. Moreover, the functional ability for more physically and cognitively structured activities was evaluated by using the 11-items version of the instrumental activities of daily living (IADL) scale. Self-sufficiency corresponded to the maximum score in both BADL and IADL scales, respectively.

#### 2.2.4. Cognitive and Affective Indicators

The cognitive level was measured using the standardized Mini-Mental State Examination (MMSE) test [37] adjusting for age, sex, education and disability [38]. An adjusted score lower than 24 was used to define dementia. Current symptomatic depression was evaluated through a short (15-item) version of the Geriatric Depression Scale (GDS-15) [39,40]. A score greater than 5 was arbitrarily selected as indicative of depression.

#### 2.2.5. Physical Performance Tests

A short battery of physical performance tests already used in longevity studies was employed according to Guralnik et al. [41]. Briefly, study participants were timed when walking a 4-m distance at their usual speed (using an ambulatory device if necessary). Next, the capacity to maintain standing balance in progressively challenging positions (tandem, semi-tandem and side-by-side stands) was tested. Finally, the ability to stand up from a chair at least five times as quickly as possible was measured. The walking endurance of participants was assessed asking them to walk a 400 m distance. A composite score from 1 to 4 was used to quantify the number of completed tests.

#### 2.2.6. Assessment of Dietary Habits

Dietary habits were evaluated using two instruments. The first was a short validated qualitative Food Frequency Questionnaire (FFQ) used in previous nutritional surveys concerning the LBZs [24,25]. The average intake of 15 foods (*cereals*: bread, pasta; *vegetables*: pulses, leafy greens and fruit; *potatoes*; meat: beef/pork, sheep/goat, chicken; *seafood*: mainly fish; *dairy products*: milk, soft and hard cheese; *fats*: olive oil and lard kept separate; *sweets*: usually traditional homemade dry cookies; coffee) over the past year was estimated. Food frequency was coded into five categories: never/rarely, 2–3 servings/month, 1–2 servings/week, 3–5 servings/week, every day, without specifying the serving size. The same questionnaire was used to recall the intake frequencies at the age of 30 years, in order to estimate the dietary intake before the NT and, in turn, highlight gross dietary changes occurring over the participant’s lifetime. As the majority of participants were born in the early 1920s, the period tested corresponded approximately to the WWII postwar period, on the edge of NT. This second questionnaire was considered a priori less accurate than the first one, given the possible failing memory of respondents for such remote events, therefore one of the family members was also interviewed to ensure consistency. Questionnaires showing concordance of answers between respondents and their family members inferior to 80% were excluded from the analysis.

### 2.3. Ethical Considerations

An Institutional Review Board approved the study protocol (131/2019/CE). All participants provided their written informed consent.

### 2.4. Statistical Analysis

All statistical analyses were carried out using SPSS statistical software (version 22.0, Chicago, IL, USA). Categorical variables were presented as absolute and relative frequencies, ordinal variables as ranks. The comparison of ordinal variables between subgroups was performed using the Mann-Whitney *U* test for independent samples or the Wilcoxon signed-rank test for paired samples. Correlation analysis was performed by calculating the non-parametric Spearman correlation coefficient. In general, a correlation coefficient of 0.3 is considered sizeable for the null hypothesis [42], therefore a minimum sample size of 84 was needed to achieve correlation coefficient of at least 0.3 with 80% statistical power [43]. Due to the potential sex difference, analyses were performed separately in males and females.

## 3. Results

Among all living subjects born on the six selected villages that are included in the Sardinian LBZ, one hundred fifty subjects aged 90 and older accepted to be interviewed, 61 of which (41%) were male. The sociodemographic characteristics of the participants have been reported previously [29] and are briefly summarized in Table 1.

### 3.1. Assessment of the Current Diet

**Figure 2:**
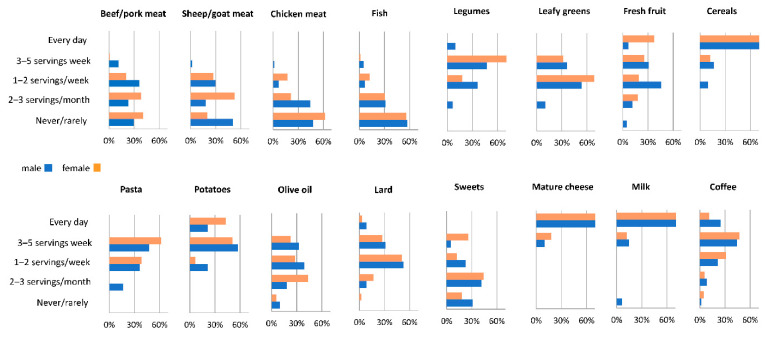
Percentage frequency of consumption of 16 food items among the study participants, reported in the x axis.

The Figure 2 shows the consumption frequency of foods separately in males and females. A strong consumption of cereals (mainly homemade bread) and, less frequently, vegetables (pulses, leafy greens) and fresh fruit was detected in both sexes. The consumption of potatoes was quite high (21% of males and 43% of females reported a daily consumption). Meat was consumed moderately, i.e., by 20–60% of female and 30–60% of male responders, and depending on the type of meat they claimed never or rarely ate it. As for beef or pork, it was consumed 2–3 servings a month by 38% of females and 1–2 servings a week by 36% of males. Sheep or goat meat was consumed at most 1–2 times a week by 27% of females and 30% of males. The consumption of poultry meat was greater: 40% of females and more than half of males consumed it at least 1–2 times a week (see Supplementary Materials). Noticeably, most LBZ families used to raise free-range chicken in the courtyard (without antibiotics and/or artificial feed). The consumption of lard was slightly lower to that of olive oil. Although BZ villages are not far from the sea, fish was not the preferred food in this community (more than half of responders never or rarely ate seafood) (Figure 2)

For each food, the mean rank per consumption category was calculated separately for males and females and reported in Table S1 of the Supplementary Materials. Significant differences between males and females were detected only for beef/pork or sheep/goat meat (consumed more frequently by males), pulses, fresh fruit, bread and pasta, potatoes and sweets (consumed more frequently by females). Some male responders also reported the occasional intake of liquor (the local grappa: filuferru) as a digestive or to make a toast with friends.

### 3.2. Dietary Intake at Age 30 and Changes across Nutrition Transition

Among 150 FFQs, a total of 98 (65.3%) were considered fully reliable as an estimate of the pre-transitional frequency of consumption of the interviewees at the age of 30. The socio-demographic features and functional status indicators of the excluded subjects did not differ significantly from those of the subjects included in the analysis.

In Table 2, the average values of the ranks for each food are listed.

Significant dietary changes were detected between the interviewees’ youth (pretransitional) and the present time (post-transitional).

To facilitate the interpretation of the results, some foods (e.g., meat) have been pooled into broad categories. For some foods the consumption frequency increased during the interviewees’ life, for example, meat in general (Z = −7.39, *p* < 0.0001), and especially beef meat (Z = −6.46, *p* < 0.0001), goat and sheep meat (Z = −4.11, *p* < 0.0001), and, less, chicken (Z = −3.21, *p* = 0.001). Similarly, fish (Z = −2.89, *p* = 0.004), fresh fruit (Z = −7.08, *p* < 0.0001), pasta (Z = −2.52, *p* = 0.012), sweets (Z = −2.69, *p* = 0.007) and milk (Z = −4.33, *p* < 0.0001) consumption increased. For pulses, vegetables and lard, the frequency decreased. The consumption of the other foods remained substantially unchanged compared to the pre-transitional period. Figure S1 of the Supplementary Materials illustrates the average rank values for every single component as well as the total difference, separately in males and females. Since the FFQ did not allow quantitative assessments, an estimate of the global energy intake was based exclusively on historical data available in the literature. For instance, in 1947 Brotzu reported an energy intake of approximately 7.54 MJ (1800 kcal/d) in Ogliastra, whereas in 1952 it had raised to 10.04 MJ (2400 kcal/d) [44].

### 3.3. Health Indicators

The mean values of the health indicators are illustrated in Table S2 of the Supplementary Materials, separately in men and women. As expected, self-perceived health was better in men despite a higher comorbidity (CIRS score). Similarly, mobility and hearing functionality were also superior in males compared to females. The remaining indicators did not show significant differences between the two sexes.

### 3.4. Association between Current Food Frequencies and Health Indicators

**Figure 3:**
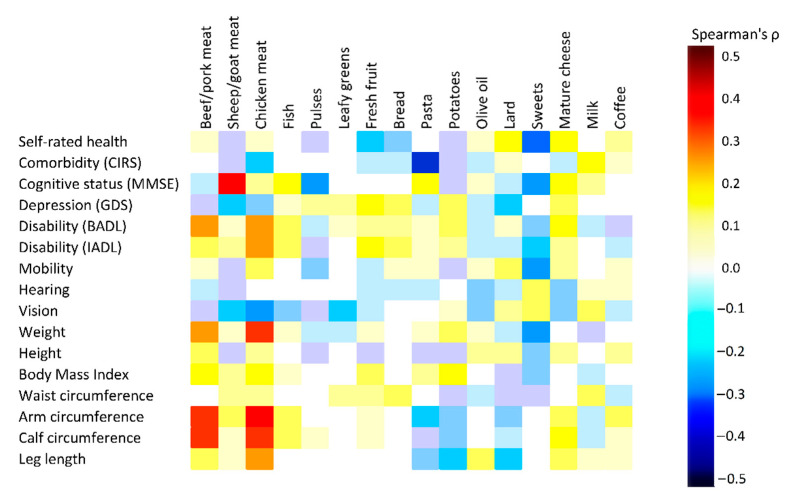
Spearman correlation heat map for the association between food frequency and health indicators.

The association of current frequencies of food consumption with health indicators was reported in Figure 3 and Table S3 of the Supplementary Materials. Briefly, the consumption of fresh fruit was associated with better functional capacity in activities of daily living (BADL: ρ = 0.217, *p* = 0.008; IADL: ρ = 0.199, *p* = 0.015). Olive oil intake was associated with better hearing and visual functionality (ρ = −0.152, and ρ = −0.145, n.s., respectively), the consumption of mature cheese was positively associated with self-rated health (ρ = 0.209, *p* = 0.010), cognitive function (ρ = 0.230, *p* = 0.012), autonomy in daily living (ρ = 0.224, *p* = 0.006) and greater calf circumference (ρ = 0.215, *p* = 0.008).

Meat consumption, especially chicken, was associated with a lower number of diseases (ρ = −0.230, *p* = 0.005), a better affective status (ρ = −0.181, *p* = 0.032), physical performance (ρ = 0.162, *p* = 0.047) and higher limb circumferences, but also with an increased BMI.

### 3.5. Association of Dietary Changes Across NT with Health Indicators

**Figure 4:**
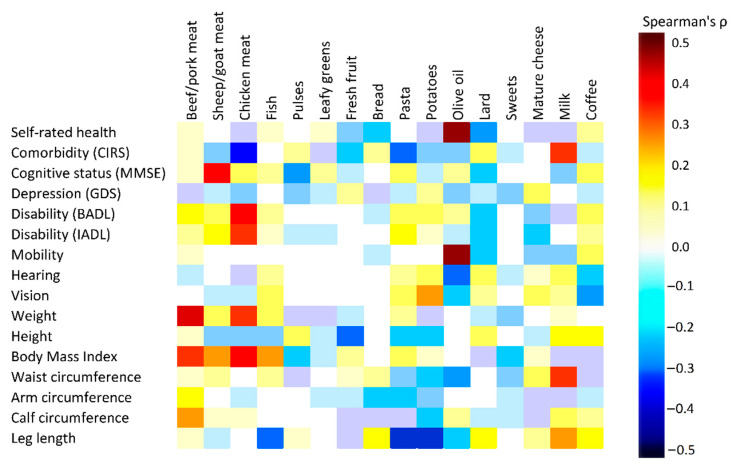
Spearman correlation heat map for the association between variation of food frequency across nutrition transition and health indicators.

The Spearman correlations between foods frequency changes and a panel of health indicators is illustrated in Figure 4 and Table S4 of the Supplementary Materials. For the overall indicators of health, the major significant positive correlation was detected between self-rate health and increased consumption of olive oil (ρ = 0.519, *p* < 0.0001), and negative with lard (ρ = −0.249, *p* = 0.030). The CIRS score correlated positively with milk (ρ = 0.300, *p* = 0.008) whereas the association was inverse (the number of diseases increased), with pasta (ρ = −0.331, *p* = 0.003) and chicken meat (ρ = −0.424, *p* < 0.0001) consumption. Cognitive status (measured through MMSE score) was positively associated only with sheep/goat meat (ρ = 0.358, *p* = 0.006), while it was negatively associated with pulse consumption (ρ = −0.259, *p* = 0.050) albeit not significantly. A higher GDS score (associated with a worse affective status) was inversely correlated with chicken meat consumption (ρ = −0.186), again without reaching statistical significance. The score for basic activities of daily living significantly improved with meat intake (ρ = 0.208, *p* = 0.025; ρ = 0.175, n.s.; and ρ = 0.351, *p* = 0.002, for beef/pork, sheep/goat and chicken, respectively). In the case of more demanding instrumental activities, a positive association was observed with sheep/goat meat (ρ = 0.229, *p* = 0.047) and chicken meat (ρ = 0.333, *p* = 0.003), and negatively with lard (ρ = −0.236, *p* = 0.040) and mature cheese (ρ = −0.237, *p* = 0.039). Better scores in physical performance tests were detected only in association with olive oil (ρ = 0.502, *p* < 0.0001) intake. As for vision and hearing, an inverse association was observed with olive oil (i.e., a greater consumption resulted in less incapacity), and for vision only, a direct association with potato consumption. The associations of dietary intake with anthropometric indicators were more complex and can be summarized as follows: weight, height and BMI correlated above all with meat and fish intake. Limb circumferences were strongly correlated with beef meat, and for calf circumference only, inversely with potato consumption. In the Supplementary Material these correlations are reported separately in males and females.

## 4. Discussion

This study aimed to investigate the evolution of dietary pattern across NT in the oldest subjects living in a secluded area of Sardinia where an exceptional proportion of individuals who become centenarians was documented [13,14,15]. Although a number of studies investigated the role of nutrition on longevity both in general [7] and in the LBZs in particular [45], detailed information addressing the relationship between NT and health status is still scarce. The present study tried to fill this knowledge gap. Before the NT, and up to the 1950s, the LBZ diet involved a mild consumption of home-processed meat which was usually salt-preserved owing to the unavailability of refrigerators [46]. It is known that a high intake of salt-preserved foods is a significant risk factor for gastric cancer [47]: accordingly, we recently reported the presence of a gastric cancer hot spot in the LBZ area [48]. Moreover, animal farming, widespread in the LBZ, was frequently reported as a risk factor for H. pylori infection, the major trigger for gastric cancerogenesis [49,50].

Regarding the current LBZ diet, our results documented a large use of olive oil, fresh fruit and cereals, considered symbolic of the Mediterranean diet, while the preference for pulses and leafy greens was lower. In addition, the analysis highlighted the consumption of products of animal origin including beef, pork and chicken meat and dairy products.

The comparison between the current diet of participants with the one dating approximately 70 years before the interview (within the limits of a memory-reconstructed diet) [51], highlighted changes in part similar to those documented in other Mediterranean regions [52]. More specifically, an increased energy intake from meat and, to a lesser extent, fish, although sea food consumption remained lower than in the villages closest to the sea. In this geographic area characterized by sheep/goat-rearing economy, dairy products intake, particularly for soft sour cheese (casu ajedu) and ricotta was high [27]. The consumption of dairy products remained constant, whereas lard decreased significantly [26]. In contrast, the analysis showed a significantly increased intake of fresh fruit and a decreased consumption of pulses and greens during NT, in contrast to the trend observed in other Mediterranean countries [50]. Sweets consumption was rare, while coffee consumption was remarkably high [27].

Furthermore, we observed a rise in the consumption of dried pasta and sweets. As a pastoralist population, the diet of participants was deeply influenced by animal breeding, implying a relatively higher consumption of cheese and lard rather than plant-based foods [29,53]. The dietary shift induced by NT, propelled by the rising economic well-being, led this population of shepherds to consume a greater amount of meat (while before the NT animals were considered a source of income and therefore rarely slaughtered) and dried pasta (regarded as a more prestigious food), as well as fewer vegetables (reputed as a poor food no longer compatible with the improving economic conditions) [29].

The change in the diet habits across the NT resulted in different outcomes related to indicators of health and functional status used in the study. As expected, the analysis showed a beneficial health effect of the increased olive oil intake on self-perceived health, physical performance and sense organs functionality. In addition, a positive association was detected between an increased sheep/goat and chicken meat intake and functionality in activities of daily living, suggesting that a surplus of animal proteins may have indirectly improved motor performance by preserving muscle mass [54]. Although meat consumption has been linked to increased all-cause mortality [55], this is restricted to red and processed meat, while the consumption of poultry meat, as in the case of LBZ, can provide several health benefits [56]. The consumption of dairy products, similar before and after the NT, did not affect health indicators.

Contrary to expectations, no significant association was noted between health indicators and fish consumption, considered a non-traditional food [29]. Pulses and greens showed a weak correlation with the health indicators of study participants. The intake of potatoes was elevated in the LBZ population before NT but largely decreased thereafter, with minimal influence on self-rated health, daily performance or motor skills, as previously reported [24]. The majority of associations were similar in the two sexes, however those of self-perceived health and consumption of fish were significant only in males (M: ρ = 0.429; F: ρ = −0.030) while consumption of chicken meat and comorbidity was significant only in females (M: ρ = −0.308; F: ρ = −0.494). The matriarchal structure of the typical shepherd LBZ families, as well as their mutual interconnections within the LBZ villages, probably accounts for most of dietary diversity between males and females.

The characteristics of the “Sardinian LBZ dietary model” that appear to be different from the Mediterranean diet canons, and now generally considered “detrimental”, may have helped in the past the islanders to survive periods of severe nutritional precariousness. In other words, they may be interpreted as a “relic of the past”, making these elders as the last survivors of generations whose food habits were shaped by an economy dominated by pastoralism and its food constraints.

Nutrition transition is often considered a negative process involving the switch from a “healthy” traditional diet rich in nutrients, to a diet characterized by excessive consumption of proteins, saturated fats and simple sugars. However, in the Sardinian LBZ the process brought also positive elements. The traditional diet, containing an unbalanced meat and dairy products intake, was progressively replaced by a diet more representative of the typical Mediterranean model, where the consumption of fish, leafy greens and fruit was higher. These positive changes, however, did not have enough influence on health indicators, perhaps because they have occurred too late in the study participants.

## 5. Strengths and Limitations

Several limitations of this study deserve to be mentioned. The most obvious is the lack of a causal correlation with the longevity of the BZ elders, being a merely descriptive study. However, the use of validated and objective methods, rather than extrapolating from proxy measures, make the results valuable. Secondly, the sample enrolled is relatively small, although the inclusion of about 50% of the eligible LBZ nonagenarians may be considered a strength. Given the difficulty to interview oldest-old subjects, a selection bias cannot be excluded entirely, although the demographic features of the nonparticipants were similar to the ones of the studied cohort [29]. Third, the temporal analysis was probably influenced by the accuracy and reliability of individual recalls, although we tried to circumvent this problem by checking with a family member the capacity of the interviewee to give reliable answers. Fourth, the FFQ used was a qualitative tool and did not allow to estimate the daily calorie intake or to demonstrate any kind of calorie restriction, even though the relatively high frequency of overweight detected suggests a positive energy balance in most respondents. Lastly, although data on income resources were collected in this rural population, socio economic status was not discussed in this survey as it had been previously reported [15,29].

Finally, it appears that our results partially disagree with the current concept of the Mediterranean diet as a largely plant-based diet. However, the real Mediterranean diet shows a wide heterogeneity in relation to the specificity of population needs and their ecosystems, and deserve to be further investigated. Nonetheless, a strength of our study is to describe for the first time the dietary pattern of the oldest subjects of the Sardinian LBZ before the NT era and nowadays, and the plausible association of dietary changes with the current health status of these agers.

## 6. Conclusions

The current diet of LBZ nonagenarians includes an adequate amount of foods typical of the Mediterranean diet, although some ancient eating habits persist, mainly determined by the local customs of a population that up to now owes its livelihood to animal farming. The increased intake of locally produced meat and olive oil in the post-NT era seems to be associated with improvement of health indicators and anthropometric parameters, suggesting that a supply of protein, healthy fats and antioxidants, may have been crucial to maintaining adequate functional capacity.

## Figures and Tables

**Table 1:** Demographic features of study participants.

Variables	Male (*n* = 61)	Female (*n* = 89)
*Age at recruitment (years)*		
Mean and standard deviation	94.2 ± 3.3	93.4 ± 4.3
Range	90–101	90–101
*Marital status, n (%)*		
Single	8 (13.1)	13 (14.6)
Married	33 (54.1)	13 (14.6)
Widowed	19 (31.1)	63 (70.8)
Divorced	1 (1.6)	0 (0.0)
*Education (years)*	3.2 ± 1.7	5.4 ± 2.1
*Living conditions, n (%)*		
Home	60 (98.4)	88 (98.8)
Nursing home	1 (1.6)	1 (1.2)
*Body mass index (kg/m^2^)*		
<18	3 (4.9)	4 (4.5)
18–24.9	31 (50.8)	49 (55.1)
25–29.9	18 (29.5)	30 (33.7)
≥30	9 (14.8)	6 (6.7)
*Activities of daily living*		
Severe disability	9 (14.7)	20 (22.5)
Moderate or no disability	52 (85.2)	69 (77.5)
*GDS* ^1^ *score*		
<6	49 (80.3)	59 (66.3)
≥6	12 (19.7)	30 (33.7)
*MMSE* ^2^ *score*		
<24	32 (52.5)	52 (58.4)
≥24	29 (47.5)	37 (42.6)

^1^ GDS, Geriatric Depression Scale [39]; ^2^ MMSE, Mini-Mental State Examination [37].

**Table 2:** Food frequency consumption before (at the age of 30 years) and after (currently) the nutrition transition.

Foods	Diet at Age 30 (*n* = 98)	Current Diet (*n* = 98)	*p* Value
Beef/pork meat	1.38	2.01	**<0.0001** ^1^
Sheep/goat meat	1.84	1.97	**<0.0001**
Chicken meat	1.55	1.58	**0.001**
*All meat*	1.79	1.85	**0.012**
Fish	1.33	1.59	**0.004**
Pulses	4.42	3.73	**<0.0001**
Greens	4.28	3.29	**<0.0001**
*Vegetables*	4.68	3.51	**<0.0001**
Fresh fruit	2.43	3.58	**<0.0001**
Bread	4.99	4.78	0.157
Pasta	3.00	3.49	**0.012**
*Cereals*	4.99	4.14	**<0.0001**
Potatoes	4.33	4.21	0.956
Olive oil	2.51	2.79	0.810
Lard	3.88	3.23	**<0.0001**
Sweets	1.88	2.29	**0.007**
Cheese	4.88	4.85	0.705
Milk	4.13	4.76	**<0.0001**
*Dairy*	4.92	4.81	**0.003**
Coffee	3.87	3.66	0.310 ^1^

^1^*p* value in bold are statistically significant.

## Data Availability

Data will be available to the reviewers upon the request.

## Supplementary Materials

The following are available online at https://www.mdpi.com/article/10.3390/nu13051495/s1, Figure S1: Average rank values at age 20 and now of single foods, separately in males and females. Table S1. Food consumption in male and female nonagenarians. Table S2. Mean value of health indicators. Table S3. Spearman’s coefficients for the association between food frequency and health indicators. Significant effects are highlighted in bold. Table S4. Spearman’s coefficients for the association between variation in food frequency and health indicators. Significant effects are highlighted in bold. Table S5. Spearman’s coefficients for the association between variation in food frequency and health indicators in males. Significant effects are highlighted in bold. Table S6. Spearman’s coefficients for the association between variation in food frequency and health indicators in females. Significant effects are highlighted in bold.
