# Supplementary material for: Evolution of the Dietary Patterns across Nutrition Transition in the Sardinian Longevity Blue Zone and Association with Health Indicators in the Oldest Old
Source: Nutrients. 2021 Apr 28;13(5):1495. doi: 10.3390/nu13051495 (PMC8146058; doi:10.3390/nu13051495)
Supplement: Supplementary file 1 [file nutrients-13-01495-s001.zip › nutrients-1167694-supplementary.pdf]

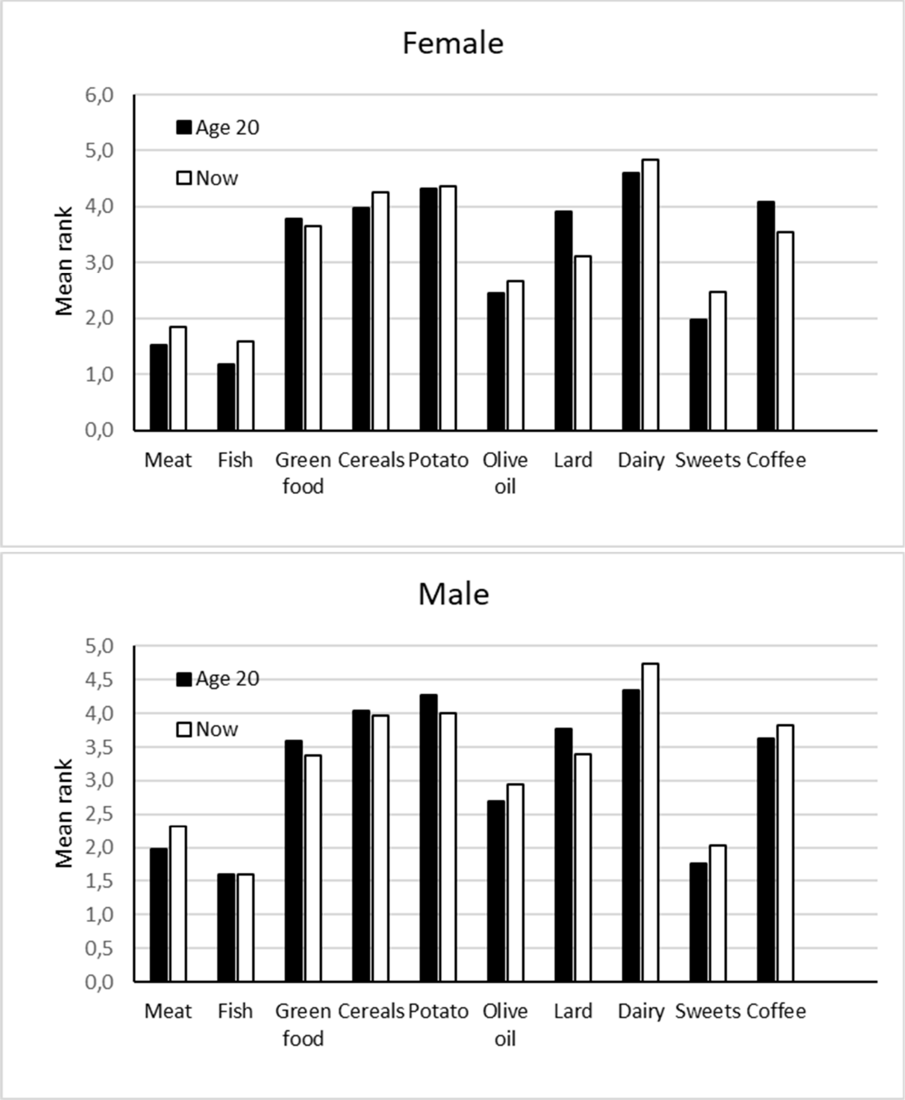
Female
6,0
5,0
4,0
3,0
2,0
1,0
0,0
Mean rank
Age 20
Now
Meat
Fish
Green food
Cereals
Potato
Olive oil
Lard
Dairy
Sweets
Coffee
Male
5,0
4,5
4,0
3,5
3,0
2,5
2,0
1,5
1,0
0,5
0,0
Mean rank
Age 20
Now
Meat
Fish
Green food
Cereals
Potato
Olive oil
Lard
Dairy
Sweets
Coffee

Figure S1

Table S1. Food consumption in male and female nonagenarians.

Foods	Mean rank		*P*-value (Mann–Whitney *U* test)
	Females (N = 89)	Males (N = 61)	
Beef/pork meat	1.82	2.30	**0.004**
Sheep/goat meat	2.07	2.50	**0.040**
Chicken meat	1.55	1.62	0.304
Fish	1.58	1.59	0.872
Pulses	3.82	3.61	**0.024**
Greens	3.31	3.26	0.777
Fresh fruit	3.82	3.23	**0.001**
Bread	4.88	4.64	**0.019**
Pasta	3.62	3.31	**0.015**
Potatoes	4.36	4.00	**0.001**
Olive oil	2.67	2.95	0.042
Lard	3.12	3.39	0.066
Sweets	2.46	2.02	**0.018**
Cheese	4.82	4.90	0.168
Milk	4.88	4.59	0.111
Coffee	3.55	3.82	0.069

Table S2. Mean value of health indicators.

**Health indicators**	Males (N=61)	Females (N=89)
**Overall**		
Self-rated health (1–5)	4.13	2.42
CIRS (0–6)	2.20	1.85
**Mental health**		
MMSE (0–30)	19.07 ± 6.61	20.28 ± 5.34
GDS (0–2)	0.50 ± 0.82	0.32 ± 0.69
**Functional capacity in activities of daily living**		
BADL (0–6)	3.79 ± 1.52	3.69 ± 1.53
IADL (0–11)	3.93 ± 3.42	3.87 ± 3.43
**Mobility**		
Composite score (0–4)	3.02 ± 0.93	2.01 ± 1.10
**Sense organs**		
Hearing score (1–4)	1.95 ± 0.92	2.31 ± 0.96
Vision score (1–4)	2.08 ± 1.11	2.60 ± 1.05
**Anthropometry**		
Weight (kg)	61.0 ± 8.1	56.6 ± 7.6
Height (cm)	163.5 ± 4.1	154.6 ± 3.0
Body mass index (kg/m^2)	22.8 ± 3.1	23.7 ± 3.3
Waist circumference (cm)	94.9 ± 9.7	100.4 ± 6.7
Arm circumference (cm)	28.1 ± 3.6	26.0 ± 3.0
Calf circumference (cm)	33.7 ± 3.1	30.9 ± 4.0
Leg length (cm)	41.2 ± 3.6	37.9 ± 2.9

* P<0.05; ** p<0.01

Table S3. Spearman's coefficients for the association between food frequency and health indicators. Significant effects are highlighted in bold.

**Health indicators**	Beef /pork meat	Sheep /goat meat	Chicken meat	Fish	Pulses	Leafy greens	Fresh fruit	Bread	Pasta	Potatoes	Olive oil	Lard	Sweets	Mature cheese	Milk	Coffee
**Overall**																
Self-rated health	0.042	–0.135	0.072	–0.001	–0.115	–0.040	–0.194	–0.151	0.012	–0.143	0.070	**0.217**	**–0.320**	**0.209**	–0.004	0.098
CIRS	0.031	–0.109	**–0.230**	0.021	0.035	0.049	–0.093	–0.070	**–0.362**	–0.135	–0.065	0.077	0.000	–0.053	**0.223**	0.064
**Mental health**																
MMSE	–0.070	**0.370**	0.118	**0.193**	**–0.251**	–0.023	–0.018	0.016	**0.223**	–0.146	0.049	–0.004	**–0.246**	**0.230**	0.108	–0.027
GDS	–0.114	**–0.199**	**–0.181**	0.068	0.143	0.101	**0.244**	**0.172**	–0.067	**0.186**	–0.046	**–0.213**	0.039	0.166	–0.011	–0.010
**Functional capacity in activities of daily living**																
BADL	**0.263**	0.080	**0.284**	0.154	–0.050	0.093	**0.217**	**0.210**	0.055	**0.178**	–0.064	0.078	–0.183	**0.224**	–0.051	–0.112
IADL	0.155	0.092	**0.245**	0.156	–0.107	–0.034	**0.199**	**0.168**	0.091	0.130	–0.047	–0.055	**–0.195**	0.128	–0.009	–0.072
**Mobility**																
Composite score	0.072	–0.102	**0.162**	0.033	–0.140	–0.009	–0.058	0.071	0.085	–0.103	0.045	**0.172**	**–0.286**	0.141	0.004	0.049
**Sense organs**																
Hearing	–0.051	–0.113	–0.035	0.040	0.048	–0.012	–0.058	–0.056	–0.089	0.007	**–0.152**	–0.089	0.160	**–0.163**	0.094	0.073
Vision	–0.101	**–0.213**	**–0.259**	**–0.172**	0.060	–0.101	**–0.204**	–0.075	0.035	0.069	**–0.145**	0.106	0.140	–0.160	0.153	–0.082
**Anthropometry**																
Weight	**0.287**	0.050	**0.302**	0.069	–0.089	–0.042	0.048	–0.012	0.088	**0.162**	0.041	–0.063	**–0.248**	0.039	–0.134	0.013
Height	**0.171**	–0.096	0.099	0.019	–0.135	0.003	–0.119	–0.025	–0.139	–0.125	0.093	0.121	–0.173	0.138	–0.020	0.139
Body mass index	**0.216**	0.118	0.229	0.053	–0.014	–0.009	**0.169**	0.040	0.137	**0.231**	–0.007	–0.112	**–0.179**	–0.037	–0.089	–0.025
Waist circumference	0.039	0.094	0.112	–0.010	0.004	0.106	0.092	0.162	0.014	–0.096	–0.046	–0.120	–0.107	0.014	0.158	–0.069
Arm circumference	**0.301**	**0.181**	**0.371**	**0.176**	0.020	–0.002	0.077	0.026	**–0.200**	–0.143	–0.017	–0.153	0.026	0.149	–0.076	**0.177**
Calf circumference	**0.308**	0.062	**0.301**	**0.179**	0.078	–0.014	0.071	0.037	–0.107	–0.157	0.003	–0.041	–0.039	**0.215**	–0.059	0.089
Leg length	**0.172**	0.068	**0.285**	–0.030	–0.027	–0.009	–0.015	–0.005	**–0.181**	**–0.230**	**0.166**	**–0.200**	–0.013	0.160	0.092	0.051

Table S4. Spearman's coefficients for the association between variation in food frequency and health indicators. Significant effects are highlighted in bold.

**Health indicators**	Beef /pork meat	Sheep /goat meat	Chicken meat	Fish	Pulses	Leafy greens	Fresh fruit	Bread	Pasta	Potatoes	Olive oil	Lard	Sweets	Mature cheese	Milk	Coffee
**Overall**																
Self-rated health	0.082	–0.036	–0.100	0.044	–0.005	0.042	–0.171	–0.196	–0.035	–0.092	**0.519**	**–0.249**	0.021	–0.095	–0.122	0.139
CIRS	0.066	–0.147	**–0.424**	–0.036	0.112	–0.119	–0.198	0.103	**–0.331**	–0.152	–0.174	0.167	–0.070	0.026	**0.300**	–0.060
**Mental health**																
MMSE	0.071	**0.358**	0.167	0.128	–0.259	0.110	–0.079	–0.006	0.177	–0.059	0.108	–0.211	–0.034	0.039	–0.172	0.173
GDS	–0.130	-0.089	–0.186	–0.035	–0.152	–0.052	0.098	–0.111	–0.069	0.102	–0.167	–0.082	–0.144	0.161	0.025	–0.074
**Functional capacity in activities of daily living**																
BADL	**0.208**	0.175	**0.351**	0.100	–0.019	–0.016	–0.002	–0.068	0.155	0.167	0.126	–0.217	0.020	–0.165	–0.100	0.150
IADL	0.116	**0.229**	**0.333**	0.047	–0.060	–0.071	–0.012	0.017	0.194	0.079	–0.057	**–0.236**	0.005	**–0.237**	0.012	0.130
**Mobility**																
Composite score	0.044	0.013	0.018	0.000	–0.038	–0.004	–0.005	–0.078	–0.029	–0.010	**0.502**	–0.198	–0.010	–0.157	–0.177	0.157
**Sense organs**																
Hearing	–0.087	–0.004	–0.118	0.122	–0.018	–0.016	–0.022	0.029	0.090	0.148	**–0.314**	0.160	–0.070	0.062	0.172	–0.204
Vision	–0.027	–0.041	–0.073	0.142	–0.006	0.015	–0.017	0.033	0.164	**0.265**	**–0.227**	0.121	–0.033	0.158	0.111	**–0.270**
**Anthropometry**																
Weight	**0.392**	**0.181**	**0.331**	0.146	–0.114	–0.082	–0.075	0.006	0.120	–0.093	–0.019	–0.058	–0.176	–0.018	0.051	0.009
Height	0.131	–0.185	–0.146	–0.178	0.158	–0.043	**–0.323**	0.015	–0.195	–0.223	0.036	0.162	0.027	–0.087	0.215	0.221
Body mass index	**0.318**	**0.268**	**0.347**	**0.244**	**–0.242**	–0.075	0.113	0.007	0.186	0.056	–0.012	–0.125	–0.207	0.058	–0.095	–0.104
Waist circumference	0.051	–0.105	0.002	0.107	–0.115	–0.030	0.066	0.137	–0.157	–0.239	**–0.276**	0.019	–0.142	0.147	**0.295**	–0.119
Arm circumference	**0.204**	0.025	–0.083	–0.017	–0.022	–0.059	–0.052	–0.211	**–0.231**	–0.150	–0.031	0.023	**0.044**	–0.139	0.093	0.063
Calf circumference	**0.277**	0.030	0.036	–0.037	0.034	–0.010	–0.132	–0.130	–0.118	**–0.229**	0.129	–0.060	–0.040	–0.103	0.159	0.121
Leg length	0.053	–0.063	–0.020	**–0.290**	**0.061**	0.029	–0.090	0.226	**–0.373**	**–0.369**	**–0.226**	**0.223**	0.013	0.106	**0.276**	**0.230**

Table S5. Spearman's coefficients for the association between variation in food frequency and health indicators in males. Significant effects are highlighted in bold.

**Health indicators**	Beef /pork meat	Sheep /goat meat	Chicken meat	Fish	Pulses	Greens	Fresh fruit	Bread	Pasta	Potatoes	Olive oil	Lard	Sweets	Mature cheese	Milk	Coffee
**Overall**																
Self-rated health	–0.257	–0.072	–0.111	**0.429**	–0.163	0.131	–0.091	–0.153	0.276	0.216	**0.683**	**–0.513**	–0.089	0.000	–0.325	–0.193
CIRS	–0.072	–0.078	–0.308	0.049	0.084	–0.028	0.030	0.211	**–0.451**	–0.176	–0.312	0.243	**–0.373**	0.044	0.314	0.137
**Mental health**																
MMSE	0.142	**0.647**	0.290	0.013	–0.401	–0.256	–0.137	–0.057	0.278	–0.183	0.071	–0.260	–0.173	0.000	–0.276	0.320
GDS	–0.121	**0.425**	**–0.429**	**–0.404**	–0.298	–0.120	0.186	–0.282	–0.299	–0.308	0.203	–0.024	–0.133	0.100	–0.130	0.110
**Functional capacity in activities of daily living**																
BADL	**0.556**	0.129	**0.387**	0.052	–0.218	–0.209	–0.114	–0.115	0.193	0.098	0.059	–0.096	–0.037	–0.299	–0.149	–0.012
IADL	**0.396**	0.160	**0.453**	–0.036	–0.317	–0.161	–0.194	0.015	0.129	–0.030	0.004	–0.044	–0.042	–0.294	–0.122	–0.050
**Mobility**																
Composite score	0.131	0.098	0.210	0.181	–0.202	–0.180	–0.271	–0.038	0.173	0.143	**0.383**	**–0.371**	–0.108	–0.264	–0.310	–0.064
**Sense organs**																
Hearing	–0.084	–0.119	–0.203	0.048	0.295	0.128	–0.075	–0.039	0.114	0.068	**–0.241**	0.291	0.083	0.209	0.244	0.228
Vision	–0.108	–0.248	–0.022	0.088	**0.376**	0.301	–0.229	–0.064	0.225	0.277	–0.272	0.185	0.249	0.177	0.247	0.117
**Anthropometry**																
Weight	**0.418**	0.276	0.245	0.297	–0.314	–0.201	–0.009	0.022	**0.327**	0.150	0.097	–0.178	–0.244	–0.166	–0.006	0.018
Height	–0.039	–0.236	0.123	0.132	–0.161	–0.212	–0.217	0.209	–0.120	0.106	–0.098	0.242	–0.042	–0.165	0.253	–0.107
Body mass index	**0.446**	**0.385**	0.200	0.225	**–0.338**	–0.186	0.061	–0.029	**0.330**	0.091	0.151	–0.268	–0.256	–0.143	–0.109	0.031
Waist circumference	0.224	–0.215	–0.087	0.001	0.164	0.201	0.163	0.075	–0.281	–0.445	–0.316	0.040	–0.224	0.451	**0.503**	–0.149
Arm circumference	0.105	–0.089	–0.285	–0.141	–0.292	–0.315	0.278	–0.259	–0.190	–0.086	–0.104	0.036	0.148	–0.216	–0.166	–0.147
Calf circumference	**0.460**	–0.087	0.022	–0.063	**–0.386**	**–0.361**	0.242	–0.074	–0.110	–0.128	0.057	–0.122	–0.060	–0.114	0.107	0.045
Leg length	–0.061	–0.029	–0.063	–0.217	–0.165	0.008	0.136	**0.387**	**–0.439**	**–0.577**	**–0.319**	0.310	–0.085	0.228	0.266	**0.194**

Table S6. Spearman's coefficients for the association between variation in food frequency and health indicators in females. Significant effects are highlighted in bold.

**Health indicators**	Beef /pork meat	Sheep /goat meat	Chicken meat	Fish	Pulses	Greens	Fresh fruit	Bread	Pasta	Potatoes	Olive oil	Lard	Sweets	Mature cheese	Milk	Coffee
**Overall**																
Self-rated health	0.100	0.254	0.166	−0.030	−0.080	0.091	0.101	0.074	0.076	0.101	**0.445**	**−0.389**	0.029	−0.033	−0.148	0.208
CIRS	0.081	−0.144	**−0.494**	−0.061	0.079	−0.135	−0.264	0.050	−0.180	−0.085	−0.121	0.080	0.124	0.048	0.265	−0.193
**Mental health**																
MMSE	0.101	0.292	0.061	0.177	−0.145	0.218	−0.112	0.096	0.110	−0.026	0.111	−0.228	−0.023	0.027	−0.097	0.113
GDS	−0.069	0.072	−0.255	0.033	0.076	−0.076	−0.252	0.081	−0.196	−0.062	**−0.475**	0.045	−0.147	0.137	0.330	0.005
**Functional capacity in activities of daily living**																
BADL	0.104	0.170	0.274	0.094	0.197	0.083	−0.045	0.036	0.043	0.229	0.170	−0.258	0.031	−0.101	−0.035	0.213
IADL	0.032	0.261	0.188	0.086	0.190	−0.032	0.013	0.061	0.181	0.135	−0.096	**−0.325**	0.043	−0.201	0.182	0.208
**Mobility**																
Composite score	−0.036	0.033	0.013	0.020	−0.003	0.129	0.280	0.092	−0.037	0.172	**0.577**	−0.182	0.013	−0.090	−0.173	0.149
**Sense organs**																
Hearing	−0.043	−0.137	−0.164	0.108	−0.142	−0.100	−0.160	−0.021	−0.112	−0.030	**−0.332**	0.217	−0.138	−0.054	0.241	−0.292
Vision	0.053	−0.133	**−0.311**	0.076	−0.101	−0.176	−0.158	0.016	−0.039	0.047	−0.169	0.208	−0.149	0.120	0.131	**−0.330**
**Anthropometry**																
Weight	**0.337**	0.216	**0.446**	0.084	0.001	−0.018	−0.070	0.049	0.036	−0.096	−0.129	−0.054	−0.153	0.077	0.060	−0.092
Height	−0.010	0.141	−0.062	−0.136	0.197	0.213	0.005	0.080	0.162	0.229	−0.018	−0.101	0.018	0.084	0.028	0.046
Body mass index	**0.347**	0.148	**0.429**	0.168	−0.074	−0.103	−0.022	−0.065	−0.078	−0.194	−0.092	0.096	−0.155	0.051	−0.020	−0.051
Waist circumference	0.049	−0.157	−0.031	0.126	−0.125	−0.111	0.001	0.093	−0.124	−0.230	−0.200	0.006	−0.044	0.030	0.263	−0.087
Arm circumference	**0.184**	**0.309**	**0.275**	0.187	0.067	0.160	−0.115	0.087	0.048	0.080	0.023	−0.142	−0.039	−0.081	0.212	0.012
Calf circumference	**0.208**	**0.319**	0.260	0.275	0.128	0.214	−0.045	0.102	0.093	−0.038	0.145	−0.188	−0.144	−0.049	0.051	0.069
Leg length	0.018	0.141	**0.313**	−0.150	0.136	0.119	0.011	−0.012	−0.008	0.132	−0.199	−0.084	0.088	0.109	0.175	0.123
